# Urethral diverticulum: A systematic review

**DOI:** 10.1080/2090598X.2019.1589748

**Published:** 2019-04-08

**Authors:** Alyssa K. Greiman, Jennifer Rolef, Eric S. Rovner

**Affiliations:** Department of Urology, Medical University of South Carolina, Charleston, SC, USA

**Keywords:** Urethral diverticula, Urethral diverticulectomy, Periurethral glands, Urethral reconstruction

## Abstract

**Objective**: To present a review of the current literature regarding the presentation, diagnosis, and treatment of female urethral diverticula (UD).

**Methods**: A systematic search of the PubMed database was performed to identify studies evaluating female UD. Article titles, abstracts and full-text manuscripts were screened to identify relevant studies, which then underwent data extraction and analysis.

**Results**: In all, 50 studies evaluating the presentation, diagnosis and treatment of female UD were deemed relevant for inclusion. Almost all studies were retrospective single-arm case series. Female UD are outpouchings of the urethral lumen into the surrounding connective tissue. The presentation of female UD is diverse and can range from incidental findings to lower urinary tract symptoms, frequent urinary tract infections, dyspareunia, urinary incontinence (UI), or malignancy. Repair of UD begins with an accurate assessment and diagnosis, which should include adequate radiographic imaging, usually including magnetic resonance imaging. Once the diagnosis is confirmed, the usual treatment is surgical excision and reconstruction, most often through a transvaginal approach. The principles of transvaginal urethral diverticulectomy include: removal of the entire urethral diverticulum wall, watertight closure of the urethra, multi-layered and non-overlapping closure of surrounding tissue with absorbable suture, and preservation or creation of continence. Results of surgical repair are usually excellent, although long-term recurrence of these lesions may occur. Complications of urethral diverticulectomy include urethrovaginal fistula, UI, and rarely urethral stricture.

**Conclusion**: Whilst urethral diverticulectomy excision and reconstruction is a challenging procedure, it is ultimately satisfying for the patient and the surgeon when relief of bothersome symptoms is achieved. Adherence to principles of reconstructive surgery is important to ensure a satisfactory result.

**Abbreviations:** PRISMA: Preferred Reporting Items for Systematic Reviews and Meta-Analyses; UD: urethral diverticulum/diverticula; UI: urinary incontinence; US: ultrasonography; VCUG: voiding cystourethrogram

## Introduction

The diagnosis and management of urethral diverticula (UD) present a challenge to the reconstructive urologist. The first challenge lies in the diagnosis of this condition, as UD present in a myriad of ways including: asymptomatic and incidentally found lesions, painful vaginal masses, bothersome LUTS, stones, or malignancy. Fortunately, the development of imaging modalities such as ultrasonography (US) and MRI has improved our understanding of and ability to diagnose UD. Once the diagnosis is made, the second challenge becomes definitive therapy, which most commonly consists of surgical excision and reconstruction. Successfully surgical management requires knowledge of the relevant surgical anatomy, as well as creativity and occasionally improvisation in the operating room.

## Methods

**Figure 1.**
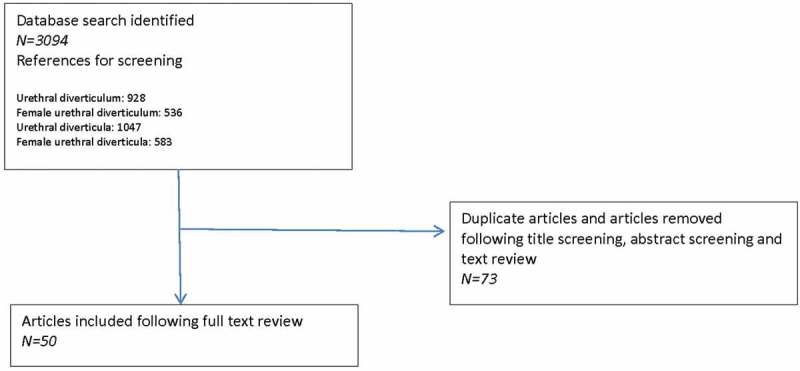
PRISMA diagram showing study acquisition.

A systematic review was performed of female UD, with an attempt to adhere to Preferred Reporting Items for Systematic Reviews and Meta-Analyses (PRISMA) guidelines. As such, we performed a structured, comprehensive literature review searching the PubMed database. We retrieved citations using search combinations including ‘urethral diverticulum’ or ‘female urethral diverticulum, or ‘urethral diverticula’ or ‘female urethral diverticula’. Article titles, abstracts, and full-text manuscripts were screened to identify relevant studies. Case series with three or fewer subjects were excluded. As the majority of articles were case series, risk-of-bias assessment was not performed. A total of 50 full-text studies were included for evidence synthesis (Figure 1).

## Pathophysiology

**Figure 2.**
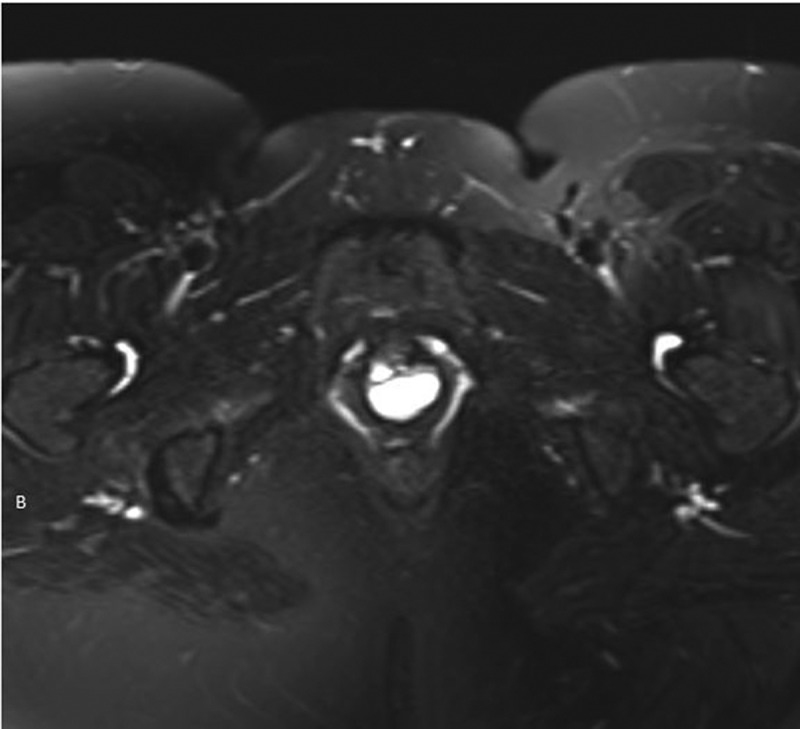
Axial T2-weighted MRI image of a 1.8-cm simple UD.

**Figure 3.**
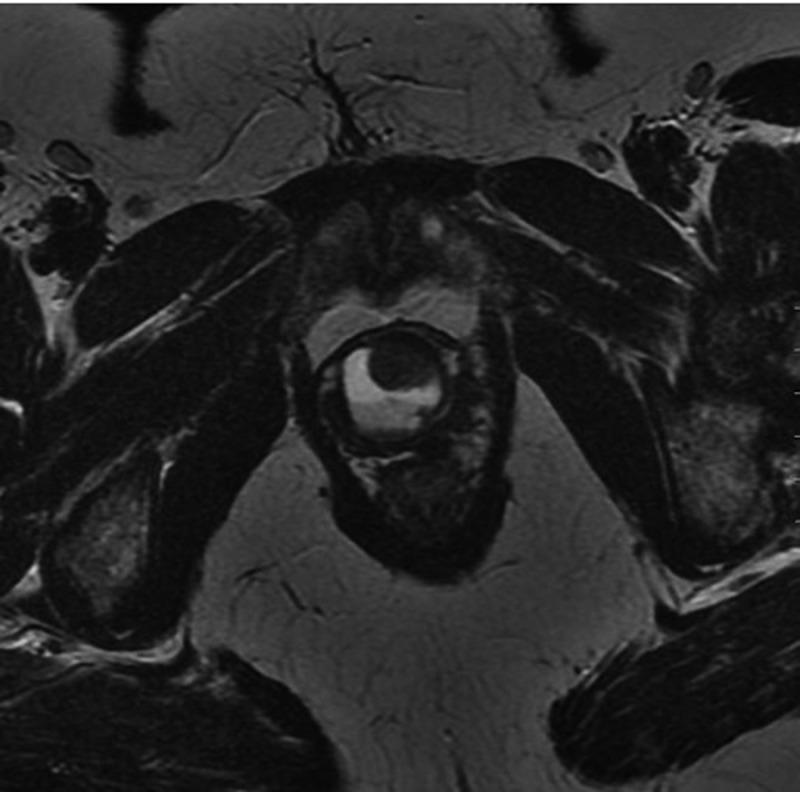
Axial T2-weighted MRI image of a 2.3-cm saddlebag UD.

**Figure 4.**
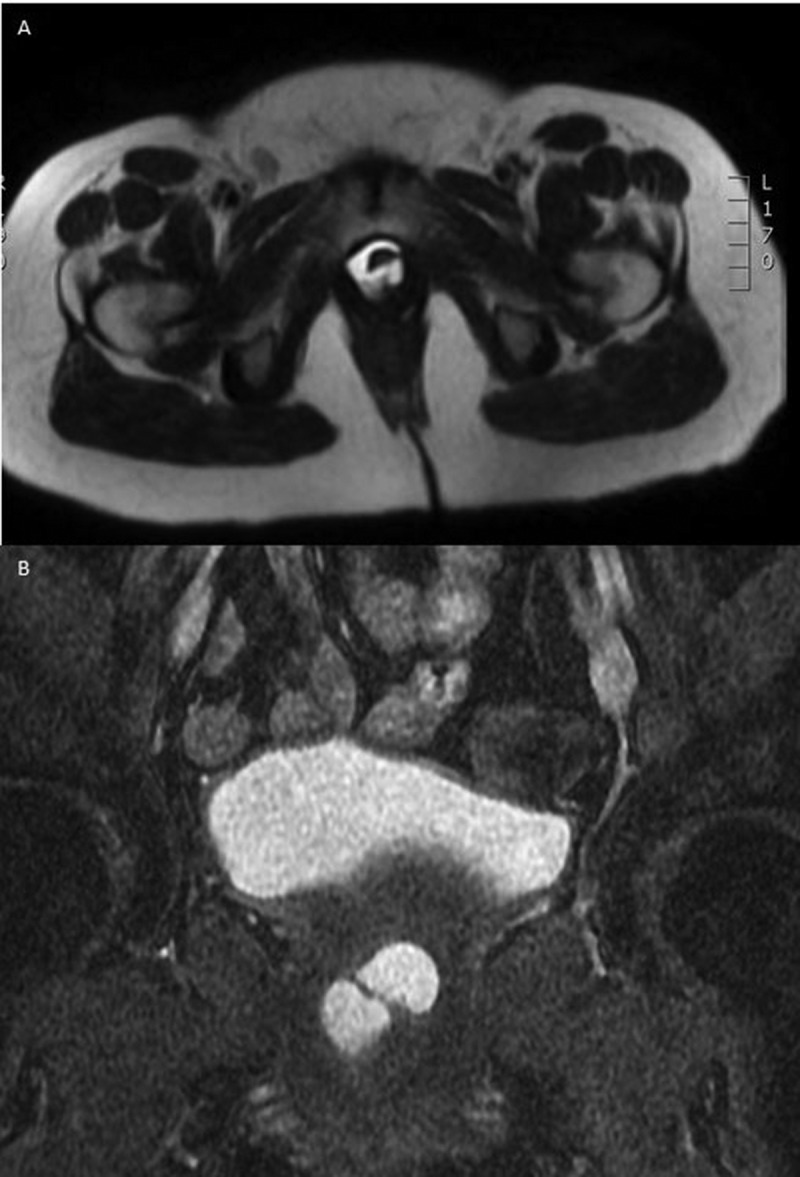
(a) Axial T2-weighted MRI image of a 3.7-cm circumferential UD. (b) Coronal T2-weighted MRI image of the same 3.7-cm circumferential UD.

UD are epithelised outpouchings of the urethral lumen into the surrounding periurethral connective tissue (also termed the periurethral fascia) [1-4]. These defects usually connect to the lumen of the urethra via a neck or ‘ostia’ and end in an isolated cyst-like outpouching. These outpouchings may be simple in nature (Figure 2), may extend partially around the urethra (saddlebag; Figure 3), or may circumferentially envelop the urethra (Figure 4) [5,6]. UD are thought to arise from repeated obstruction, infection and subsequent rupture of periurethral glands into the urethral lumen, resulting in an epithelialised cavity that communicates with the urethra [4]. Over 90% of UD ostia are located posterolaterally in the mid-to-distal urethra, a fact which is supported by the observation that the periurethral glands are located dorsolateral to the urethra, draining in the distal one-third of the urethra [7,8]. Iatrogenic damage to the urethra may also play a role, as up to 20% of women with UD are noted to have a history of prior urethral surgery, dilatation, or traumatic delivery [3].

## Prevalence and risk factors

UD is a rare condition that affects between 1% and 6% of adult women; however, as many patients are asymptomatic or misdiagnosed, the true incidence is unknown [1,2]. Most patients with UD present between the third and seventh decade of life, with a median age of 40 years, but presentation can occur at any age [9–12]. UD have been reported in 1.4% of women who present for the evaluation of urinary incontinence (UI), with the diagnosis being made in up to 80% of women who present with a periurethral mass [1–3]. Whilst iatrogenic damage to the urethra is thought to play a role in the development of UD, as well as multiparity, a recent study showed that 31% of patients with UD were nulliparous [13].

## Presentation

The presentation of UD is variable, ranging from incidental findings on physical examination or cross-sectional imaging, to frequent UTIs, dyspareunia, UI or malignancy. The classic presentation has been historically described as the ‘three Ds’: dysuria, dyspareunia, and dribbling (post-void). In truth, none of these symptoms are sensitive or specific for UD, with the classic triad only being seen collectively in 5% of patients with UD [14]. The most common presentations include vaginal mass, irritative LUTS, and recurrent UTIs [13,15,16]. Recurrent UTIs are seen in one-third of patients, likely due to urine stasis [10,13], and recurrent UTIs should trigger evaluation for a UD. Additionally, dyspareunia is noted in 12–24% of patients [13,17]. Patients may present with a palpable anterior vaginal wall mass, which upon compression may exude retained urine or debris per urethral meatus. Nonetheless, up to 20% of patients lack symptoms, with UD being an incidental finding on imaging. Somewhat surprisingly, the size and complexity of the UD does not correlate with symptoms [18]. The vague and overlapping nature of symptoms frequently delays the diagnosis of UD by 2–5 years, with the mean interval between onset of symptoms and diagnosis of 5.2 years [1,3,19,20]. Therefore, it is important to harbour a level of suspicion and perform a thorough pelvic examination in women who have LUTS, UTIs, or in whom a palpable vaginal mass is identified.

## Physical examination

When performing a pelvic examination, the anterior vaginal wall should be palpated for masses and associated tenderness. Most UD are located ventrally on the anterior vaginal wall, 1–3 cm inside the introitus, but may extend more proximally toward the bladder neck. This should be considered; as such UD will distort the bladder outlet and trigone, placing the bladder and ureters at risk during surgical excision and reconstruction. A hard anterior vaginal wall mass may indicate a calculus or malignancy within the UD, and should prompt further investigation. Whilst stripping the urethra distally may express urine or debris from the UD, this is not pathognomonic and is only present in the minority of patients [21]. Examination should include an assessment for vaginal wall atrophy, as the presence of poorly oestrogenised vaginal tissue may present challenges intraoperatively. A complete vaginal examination should include a provocative measure to elicit stress UI (SUI), an assessment for vaginal prolapse, and may include a catheterised post-void residual.

## Differential diagnosis

The differential diagnosis of periurethral masses includes: vaginal wall cysts, leiomyoma, Skene gland abnormalities, Gartner’s duct abnormalities, urethral prolapse, and urethral caruncle in addition to UD. A thorough pelvic examination with palpation of the anterior vaginal wall for tenderness or discharge may not be sufficient in making a diagnosis. In such instances, and for operative planning, further cystoscopic and radiographic evaluation is warranted.

## Urine studies

Whilst many patients are on suppressive antibiotic therapy, urine analysis and urine culture should still be performed. Historically, the most common organism isolated in patients with UD is *E. coli*, although other gram-negative enteric flora are often present [17]. In patients with severe irritative voiding symptoms, haematuria, or suspicion of malignancy, a urine cytology can be checked.

## Cystourethroscopy

**Figure 5.**
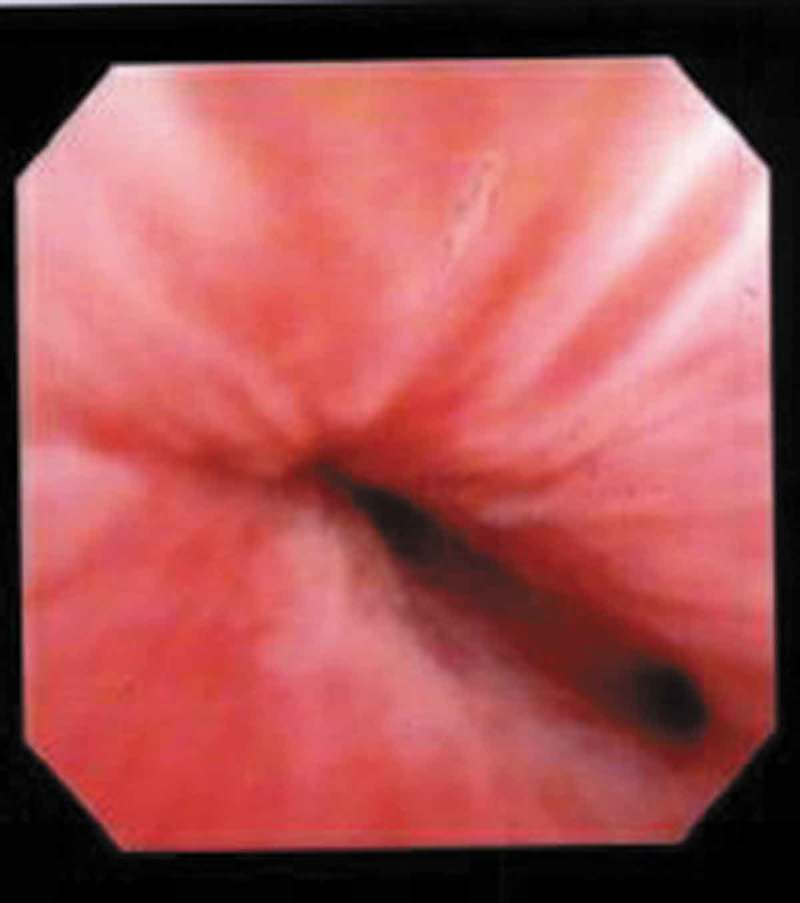
Cystoscopy with visualisation of UD ostium at the 4 o’clock position just proximal to the mid-urethral sphincter.

When UD is suspected, cystourethroscopy offers the opportunity to visualise the location of the diverticular ostium and to evaluate for other causes of irritative or obstructive voiding symptoms (Figure 5). A flexible cystoscopy or rigid female cystoscope is recommended for the evaluation of the female urethra. The short beak on the sheath of a specially designed rigid female cystoscope maintains the flow of the irrigation solution immediately adjacent to the lens and aids in distention of the relatively short (as compared to the male) urethra, permitting improved visualisation. Visualisation of the ostia is often facilitated by compressing the bladder neck whilst simultaneously applying pressure to the suspected diverticular sac by an assistant to visualise luminal discharge. Despite the use of specialised equipment and evocative manoeuvres, visualisation of the diverticular ostium on cystourethroscopy is variable and reported in 15–89% of cases [1,19,20,22,23]. Again, the ostium is usually located posterolaterally in the proximal or mid third of the urethra.

## Imaging

**Figure 6.**
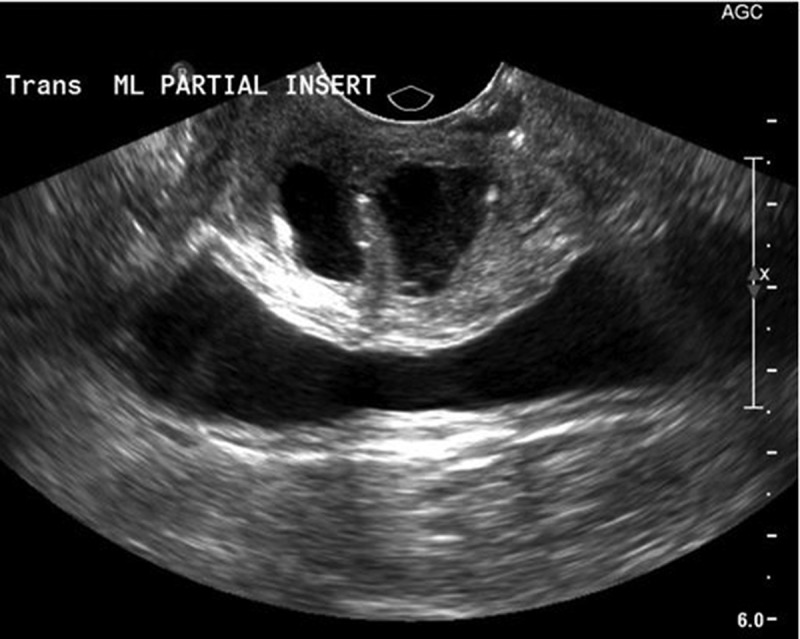
Transvaginal US of a 2.3-cm saddlebag UD.

**Figure 7.**
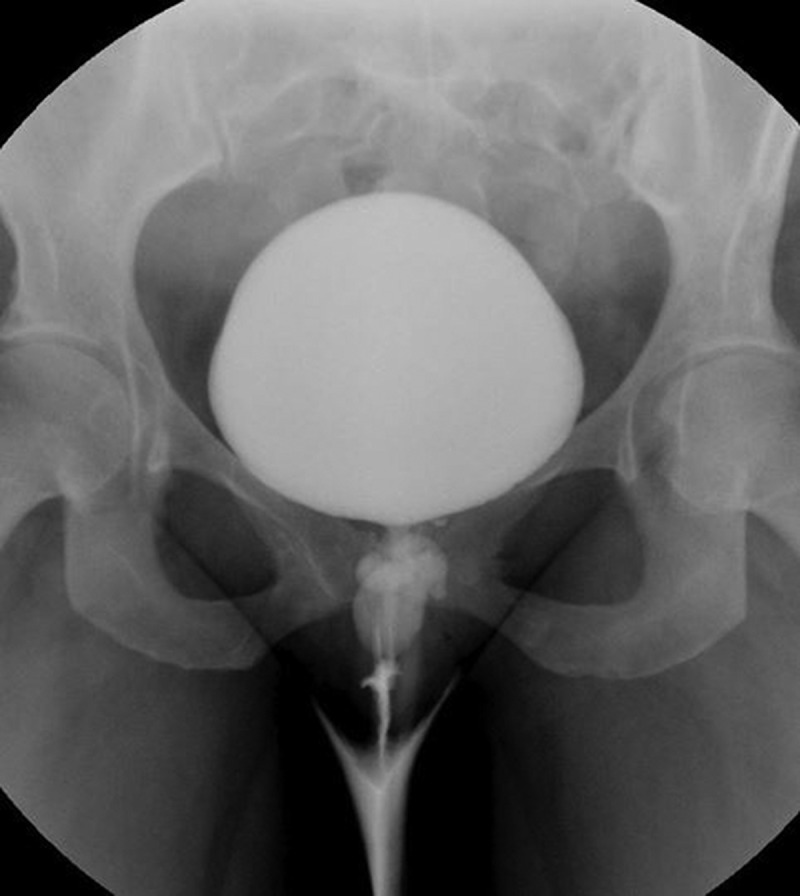
VCUG notable for a lobulated opacification inferior to the bladder, consistent with a large, multi-lobulated UD.

Radiological modalities for evaluation of UD include: US, voiding cystourethrogram (VCUG), and MRI (Table 1). Transperineal and transvaginal US have reported sensitivity of up to 95–100% and may be useful in the intraoperative identification of the UD in difficult dissections [1,19,24] (Figure 6). Whilst US is readily available and inexpensive without the need to expose the patient to radiation, it is highly operator dependent, and the sensitivity of this imaging modality is predicated on the skill of the sonographer, with some studies reporting a < 50% sensitivity in evaluation of known UD [1,3]. Newer US techniques, such as transurethral contrast-enhanced US have been reported to have a 95% sensitivity and 100% specificity in the diagnosis of UD [25]. VCUG offers the ability to visualise the UD when the ostium is patent, with a sensitivity of 67–95% [1,26] (Figure 7), but is limited by the invasive, uncomfortable nature of this study. MRI on the other hand, is the optimal study for the diagnosis and operative planning of UD. Its superb soft tissue contrast allows for accurate delineation of urethral anatomy and it’s supporting structures and has become the ‘gold standard’ for UD diagnosis. MRI is able to delineate the ostium in 85% of cases [27–29]. T2-weighted imaging will display the UD as a bright, fluid-filled entity adjacent to the urethra (Figures 2–4).

**Table 1.** Summary of imaging techniques.

Imaging technique	Pros	Cons
Cystourethroscopy	-Allows comprehensive evaluation for other causes of symptoms -no radiation exposure	-visualisation of ostium is variable (15–89%) -inability to fully characterise UD -invasive procedure
VCUG	-sensitivity of 67–95%	-invasive procedure -must void to image UD -ostia must be patent to image UD -poor stream underestimates size -radiation exposure
US	-sensitivity of 95–100% -readily available -inexpensive -non-invasive -no radiation exposure	-highly operator dependent -inability to visualise ostium
MRI	-‘gold standard’ imaging study -superb soft tissue contrast for delineating urethral anatomy -delineates ostium in 85% of cases -non-invasive -no radiation exposure	-expensive

## Urodynamics

Urodynamics may be beneficial in patients with a UD who have UI, to clarify if genuine SUI is present or if the apparent UI is actually due to post-void dribbling due to residual urine in the UD after voiding. In cases where UI is present, urodynamics may be helpful to better characterise and document the presence of genuine SUI, as well as assess for the presence of detrusor dysfunction. About 50% of women with UD will demonstrate SUI on urodynamic evaluation [10,30]. For patients undergoing surgery for UD with coexistent bothersome SUI demonstrated on physical examination or urodynamics, a concomitant anti-incontinence surgery can be offered with good success [30–33].

## Malignancy

The natural history of untreated UD is unknown. Whether such lesions enlarge, become more symptomatic or are associated with other complications such as malignant degeneration, is poorly characterised. As such, not all UD mandate surgery. However, it should be noted that up to 10% of UD show atypical pathological findings without any obvious imaging findings [18–34], with malignancy being found in 1–6% of UD [3,22,34,35]. The most common malignancies reported are adenocarcinoma, TCC (urothelial carcinoma) and squamous cell carcinoma. Patients who are not surgical candidates and those who do not desire surgical excision should be counselled as to the risk of malignant transformation and should undergo continued monitoring. If malignancy is found, pelvic exenteration, lymphadenectomy and urinary diversion is recommended when applicable.

## Indications for surgical repair

Once the diagnosis is confirmed in symptomatic patients, the treatment of UD usually consists of surgical excision and reconstruction. Indications for surgical excision and reconstruction of UD include refractory symptoms such as irritative voiding symptoms, pelvic pain, dyspareunia, and recurrent UTIs. Minimally symptomatic patients and those who desire non-operative management may be placed on antibiotic prophylaxis. In such individuals, post-void stripping of the anterior vaginal wall would be expected to empty the UD cavity and potentially reduce post-void dribbling and recurrent UTIs.

## Preoperative preparation

Prophylactic antibiotics in addition to preoperative parenteral antibiotics can be administered, especially for those with recurrent or persistent UTIs based on preoperative culture data. Application of topical oestrogen creams for several weeks before surgery may be beneficial in some patients with postmenopausal atrophic vaginitis in improving the quality of the tissues. Additionally, the importance of appropriate preoperative patient counselling to set expectations is essential, as some associated symptoms of pain, dyspareunia, voiding dysfunction, UI and recurrent UTIs may not improve or resolve with surgical management of the UD.

## Management of SUI

UD and SUI often co-exist, with reports of anywhere between 10% and 57% of patients with UD also presenting with SUI [35,36]. Only ~50% of these patients were found to have true SUI vs post-void dribbling [10]. Conversely, UD can also mask SUI due to mass effect in 10–33% of patients, especially in proximal UD >3 cm [35]. As such, there is no consensus on appropriate timing of surgical management of these two conditions. When treating concomitant UD and SUI, some surgeons favour a staged procedure, whilst others recommend simultaneous pubovaginal sling placement. Concomitant autologous pubovaginal sling placement has been found to be safe and effective for treatment of SUI at the time of urethral diverticulectomy and should be decided on an individualised basis after appropriate preoperative counselling and assessment of degree of bother of SUI [18,33,36]. The use of synthetic materials as a concomitant sling material is not recommended due to the risk of erosion of the synthetic graft [37].

## Techniques for repair

A myriad of surgical techniques to manage UD have been reported since 1805 when Hey first described transvaginal incision of the UD with packing of the cavity with lint [38]. Alternative approaches to excision and reconstruction include marsupialisation [39,40], endoscopic unroofing [41,42], fulguration [43], and incision and obliteration with oxidised cellulose or polytetrafluoroethylene [44,45]. For patients with very distal UD who do not desire extensive surgical reconstruction, marsupialisation of the UD into the vagina via a deep incision into the ventral urethra is an option (Spence–Duckett procedure) [39,40]. Patients are counselled that there is a risk of SUI, as proximal incision of the ventral urethra may result in injury of the urethral sphincter and *de novo* postoperative SUI.

Rarely, in pregnant women or in patients with severe symptoms or an infected UD in whom elective excision and reconstruction should be postponed, a transvaginal incision directly into the UD (‘diverticulotomy’) can be performed to create a temporary urethrovaginal fistula traversing the UD cavity. This decompresses the UD until elective excision and reconstruction can be performed. Such patients should be counselled that if the UD is located proximally or they have an incompetent bladder neck, they may have constant leakage of urine per vagina through the iatrogenic urethrovaginal fistula until definitive reconstruction is performed.

## Excision and reconstruction

Surgical excision of the UD with appropriate tension-free, multi-layered closure is the ‘gold standard’ treatment with most studies reporting success rates >90% [1,3,18,22,46]. Whilst some surgeons may disagree on the type of vaginal incision (midline vs inverted ‘U’ vs inverted ‘T’), whether it is necessary to remove the entire epithelialised portion of the lesion, and the optimal type of postoperative catheter drainage (urethra only vs urethra and suprapubic), overall the principles of urethral diverticulectomy are agreed upon and well-described. The technique described herein is similar to that described by Leach et al. [16].

## Surgical technique

The principles of successful transvaginal urethral diverticulectomy include: removal of the entire UD sac, watertight closure of the urethra, multi-layered and non-overlapping closure of surrounding tissue with absorbable suture to close dead space, and preservation or creation of continence.

The patient is placed in lithotomy position with standard application of vaginal antiseptic. A 16-F urethral Foley catheter is placed. Exposure is facilitated with a weighted vaginal speculum and a Scott retractor with hooks. A posterolateral episiotomy may be beneficial in some patients for additional exposure although the midurethral location of most UD usually obviate the need for this.

**Figure 8.**
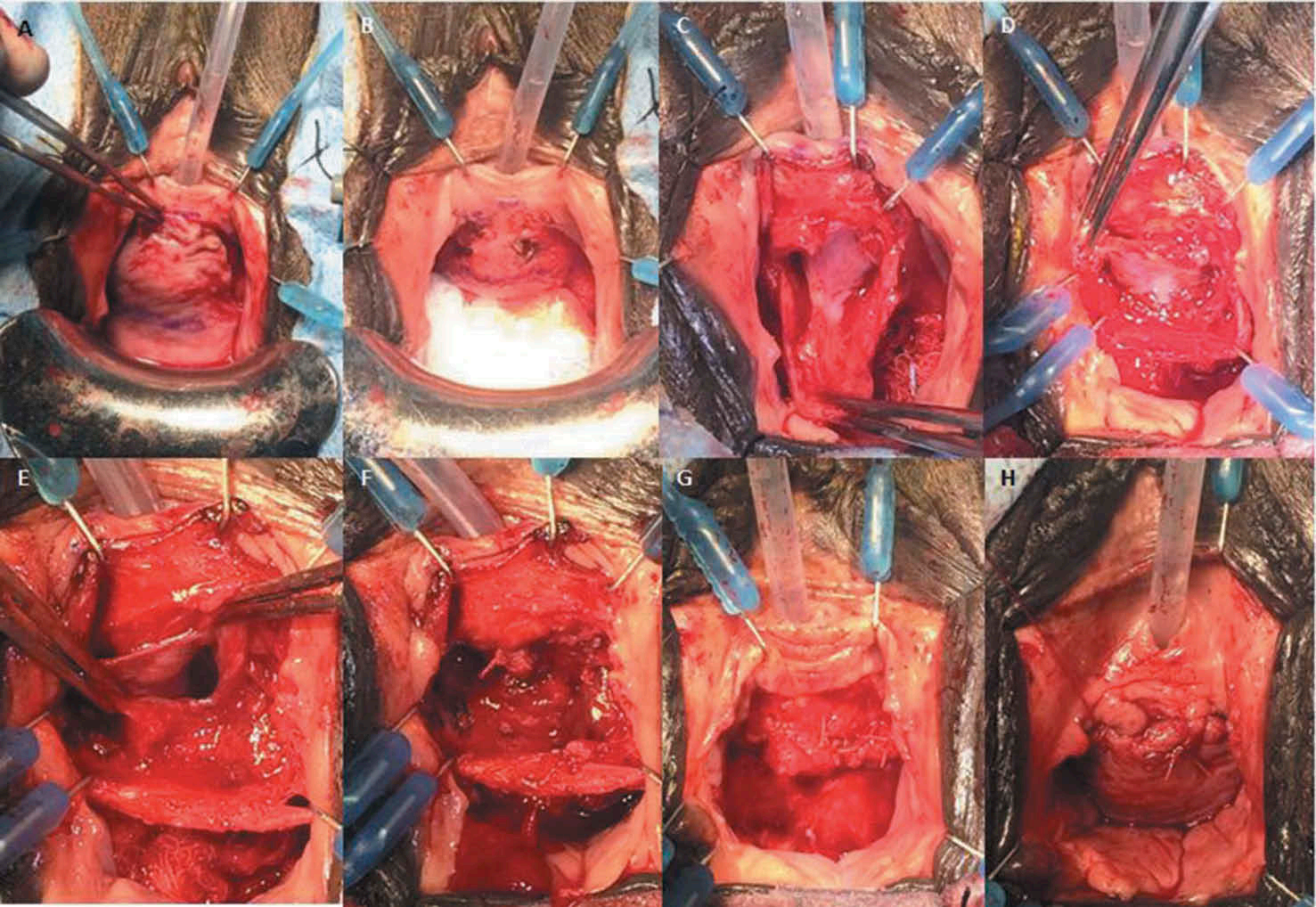
(a) An inverted ‘U’ incision is marked on the anterior vaginal wall. Retraction is accomplished with a weighted vaginal speculum and a Scott retractor. (b) After dissection of the anterior vaginal wall, which is packed away with a moist gauze, a transverse incision is made in the periurethral fascia. (c) The periurethral fascia is dissected from the underlying UD. (d) The UD is grasped and dissected circumferentially down to the ostium. (e) The UD is excised. (f) The urethra is closed with running 4/0 absorbable sutures. (g) The periurethral fascia is closed with 3/0 interrupted absorbable sutures perpendicular to the urethral closure. (h) The anterior vagina wall flap is sutured in a running-locking fashion with 2/0 absorbable sutures.

An inverted ‘U’ is marked out along the anterior vaginal wall with the base of the ‘U’ at the level of the distal urethra and the limbs extending to the bladder neck (Figure 8(a)). The limbs of the ‘U’ extend progressively more lateral as the incision proceeds proximally (toward the bladder neck) to avoid ischaemia of the distal lateral edges of the flap. This ‘U’ incision is preferred by some surgeons compared to an inverted ‘T’ incision, as it provides superior lateral exposure at the level of the mid-vagina and can be extended proximally toward the bladder neck if needed. The ‘U’ incision also minimises any overlapping suture lines at closure. To facilitate dissection, normal saline is injected along the incision line beneath the vaginal wall. Injection of vasoconstrictive agents is an alternative; however, this may mask recognition of bleeding vessels and potentially increase the risk of delayed haemorrhage.

An anterior vaginal wall flap is created by careful dissection with Metzenbaum scissors in the potential space between the vaginal wall and the periurethral fascia. Initial dissection laterally for a few millimetres from the limbs of the inverted ‘U’ incision towards the ipsilateral vaginal fornix aids in demarcation of the flap for closure later. The use of sufficient counter-traction with Allis clamps on the flap during this portion of the procedure is important to maintain the proper plane of dissection. The proper plane is identified by noting the glistening internal side of the vaginal wall flap. Care is taken to preserve the periurethral fascia and avoid inadvertent entry into the UD. Preservation of the periurethral fascia is important, as this will allow a multi-layered closure of dead space and decrease the risk of UD recurrence and fistula formation postoperatively. Pseudodiverticula have been described where periurethral fascia is attenuated or absent [47]. In these patients, an interpositional flap or pubovaginal sling may be utilised for reconstruction.

Once the anterior vaginal wall flap is dissected, it is packed cephalad with moist gauze deep in the vagina. The periurethral fascia is incised transversely over the UD and dissected down to the external UD wall (Figure 8(b)). The periurethral fascia is then dissected off of the UD circumferentially to delineate the margins of the UD, with care taken to avoid entry into the UD (Figure 8(c)); however, it may be necessary to open the UD to facilitate dissection. At this point, the UD is dissected to the ostium where it connects to the urethra (Figure 8(d)). Every effort should be made to remove the entire epithelialised surface of the UD to prevent recurrence. It is acceptable to remove a small component of inflamed or adherent urethral wall, especially at the ostium. If the ostium is difficult to locate, the location of the ostium can be identified after the UD is opened by infusing saline through an 18-F or larger angiocatheter placed into the urethral meatus adjacent to the Foley catheter. The urethra will distend upon injection and a jet of saline will be visualised at the ostium.

The Foley catheter may be seen after the UD is excised at the site where the ostium was removed (Figure 8(e)). The urethra can then be reconstructed over the Foley catheter in a watertight fashion with 4/0 synthetic absorbable sutures following standard reconstructive principles of a tension-free and watertight closure (Figure 8(f)). The periurethral fascia is then re-approximated with interrupted 3/0 synthetic absorbable sutures perpendicular to the orientation of urethral closure, with care taken to close all dead space (Figure 8(g)). In patients with poor quality tissues, attenuated periurethral fascia, or significant scarring, a vascularised adjuvant flap such as a Martius flap may reduce the risk of wound breakdown and subsequent complications such as urethrovaginal fistula. The anterior vaginal wall flap is then re-approximated with 2/0 absorbable sutures to complete a three-layer closure (four layers if a Martius flap is used) (Figure 8(h)). The Foley catheter is left indwelling and an antibiotic impregnated vaginal packing is placed after closure.

## Postoperative care

Patients are generally admitted overnight for observation and to continue parenteral antibiotics for 24-h postoperatively. The vaginal packing is removed on postoperative day one and the patient is discharged with an indwelling urethral catheter (and possibly an additional suprapubic catheter). Anticholinergics are prescribed to reduce bladder spasms, and a stool softener is given to reduce straining for bowel movements. For those patients with history of recurrent UTIs, a suppressive antibiotic is prescribed until the urethral catheter is removed. Patients then return 10–14 days postoperatively for a pericatheter VCUG and, if no extravasation is observed, the catheter is removed. If extravasation is seen, then repeat pericatheter VCUGs are performed every 1–2 weeks until resolution of extravasation. In most cases, extravasation will resolve in several weeks with conservative management [48]. Patients are instructed to avoid anything per vagina for 6 weeks.

## Outcomes

Transvaginal urethral diverticulectomy has a high success rate of between 84% and 98%, with a re-operation rate of 2–13% after primary repair during a mean follow-up of 12–50 months [1,3,18,19]. Those studies with longer follow-ups report a higher rate of recurrence, which is unsurprising. Risk factors associated with recurrence include: history of multiple UD, proximal UD, prior pelvic surgery or radiation. A recurrent UD following initial successful urethral diverticulectomy may occur as a result of a new infection or traumatic insult such as childbirth, a new UD, or recurrence of the original lesion. Recurrence of UD may be due to incomplete removal of the UD, inadequate closure of the urethra or residual dead space, or other technical factors. Repeat urethral diverticulectomy surgery represents a unique challenge due to altered anatomy, scarring, and difficulty identifying proper anatomical planes.

## Complications

Early common postoperative complications include: UTI (0–39%), *de novo* SUI (3.8–33%), and *de novo* urinary retention (0–9%), especially in the setting of concomitant placement of an autologous pubovaginal sling [1,3,18,19]. Delayed complications such as urethral stricture are reported in 0–5.2% of cases [1,18,22]. Urethrovaginal fistula is a devastating complication presenting in 0.9–8.3% of cases [49]. A distal fistula located beyond the sphincteric mechanism can present with split urinary stream or vaginal voiding and may not require repair. However, a fistula located anywhere from the mid-urethra to the bladder neck may result in UI. These patients should undergo repair with consideration of an adjuvant tissue flap, such as a Martius flap, to aid in closure. The timing of the fistula repair is not well defined, with a delay of 3–6 months after the initial repair generally being a good balance between patient discomfort and optimal tissue quality. Rare complications include: distal urethral necrosis, bladder injury, urethral injury, ureteric injury, and vaginal scarring or narrowing with consequent dyspareunia [49]. Attention to surgical technique including: preservation of periurethral fascia, a well-vascularised anterior vaginal wall flap, multi-layered non-overlapping suture lines, adequate haemostasis, and infection prevention, should minimise the potential for postoperative complications.

## Conclusion

Management of UD starts with a high index of suspicion, accurate assessment and diagnosis. Preoperative imaging with MRI aids in surgical planning and urodynamics may assist in management of patients presenting with concomitant UI. Whilst urethral diverticulectomy excision and reconstruction is a challenging procedure, it is ultimately satisfying for the patient and the surgeon when relief of bothersome symptoms is achieved. Adherence to principles of reconstructive surgery including careful dissection, preservation of the vascular supply of flaps, avoidance of overlapping suture lines, and watertight closure are important to ensure a satisfactory result.
